# Immunoconjugates in cervical cancer: a bibliometric and thematic mapping analysis of global research trends, 2015–2024

**DOI:** 10.3389/fonc.2026.1732209

**Published:** 2026-04-23

**Authors:** Xiaodong Wang, Di Xiong, Songli Cui, Bingchen Duan, Gouping Ding, Yiping Huang, Qianqian Wang

**Affiliations:** 1 Department of Oncology, Zhuzhou Hospital Affiliated to Xiangya School of Medicine, Central South University, Zhuzhou, China; 2 Department of General Medicine, Zhuzhou Hospital Affiliated to Xiangya School of Medicine, Central South University, Zhuzhou, China; 3 Department of Orthopaedic Surgery, Zhuzhou Hospital Affiliated to Xiangya School of Medicine, Central South University, Zhuzhou, China

**Keywords:** antibody drug conjugate, bibliometric analysis, cervical cancer, immunoconjugate, keyword burst

## Abstract

**Background:**

Immunoconjugates, particularly antibody drug conjugates, are emerging as clinically relevant options for recurrent or metastatic cervical cancer. A quantitative overview of how this field has evolved over the last decade remains limited.

**Methods:**

Publications from 2015–2024 were retrieved from the Web of Science Core Collection and Scopus, restricted to English articles and reviews, de-duplicated, and screened by two reviewers, yielding 244 records for analysis. Bibliometric indicators were calculated using bibliometrix in R, while VOSviewer and CiteSpace were used for keyword co-occurrence mapping and burst detection, respectively.

**Results:**

Output increased markedly after 2021, consistent with a shift toward clinical translation. Keyword mapping identified five interlinked themes spanning clinical safety and toxicity management, hematologic malignancy related translational influences, preclinical mechanisms, gynecologic oncology applications, and clinical trial design with emerging therapeutics. Burst analysis indicated a temporal transition from early model based and mechanistic terms to clinically oriented hotspots, including tissue factor and immunotherapy associated concepts.

**Conclusions:**

The literature depicts rapid maturation of immunoconjugate research in cervical cancer from foundational studies toward clinically actionable targets, safety optimization, and combination strategies, providing a roadmap for prioritizing future translational efforts.

## Graphical Abstract

**Figure:**
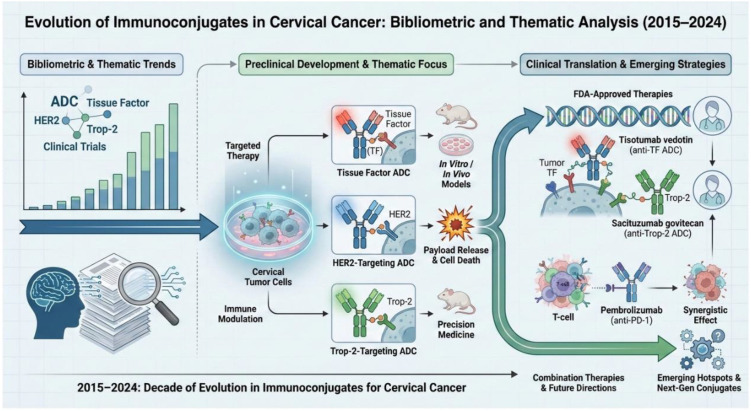
Overview of the bibliometric and thematic mapping workflow and key findings on immunoconjugate (including antibody–drug conjugate) research in cervical cancer from 2015–2024. Publications were retrieved from the Web of Science Core Collection and Scopus, screened to yield 244 records, and analyzed using bibliometrix (R) with visualization in VOSviewer and burst detection in CiteSpace. The graphic summarizes the accelerated publication growth after 2021 and the emergence of clinically oriented hotspots and interlinked themes spanning toxicity/safety, preclinical mechanisms, gynecologic oncology applications, and trial-focused translational research.

## 1 Introduction

Cervical cancer persists as a major global health concern, ranking fourth among malignancies in women, with approximately 662,000 new cases and 348,000 deaths reported in 2022 (1–3). This disease contributes substantially to the annual total of around 20 million new cancer diagnoses across populations (including nonmelanoma skin cancers) (1). Primarily driven by chronic high-risk human papillomavirus infection, cervical cancer disproportionately burdens low- and middle-income countries, where preventive strategies such as vaccination and screening remain limited (3, 4).

Despite progress in primary prevention, advanced or recurrent cervical cancer poses significant therapeutic challenges, with a five-year survival rate of about 17-20% in metastatic settings (5, 6). Standard interventions, including surgery, radiotherapy, and platinum-based chemotherapy combined with bevacizumab or immune checkpoint inhibitors like pembrolizumab, have enhanced outcomes for locally advanced disease (7, 8). However, treatment options for recurrent or metastatic cervical cancer are restricted, yielding modest response rates and limited durability, which highlights the critical need for novel therapies.

Antibody-drug conjugates represent an emerging class of targeted agents that integrate monoclonal antibodies with cytotoxic payloads through linkers, facilitating selective delivery to antigen-expressing tumor cells and reducing off-target effects (9, 10). This approach can improve efficacy over conventional chemotherapy, as evidenced in various solid tumors (11, 12). In gynecologic cancers, these conjugates target overexpressed antigens, including tissue factor, folate receptor alpha, human epidermal growth factor receptor 2, and trophoblast cell-surface antigen 2, which are commonly elevated (e.g., >80% for Trop-2 in ovarian/endometrial; 90% for TF in cervical) (13, 14).

Tisotumab vedotin, the first antibody-drug conjugate approved by the US Food and Drug Administration in September 2021 for recurrent or metastatic cervical cancer after chemotherapy progression, illustrates this advancement (11, 14). It targets tissue factor, a transmembrane protein overexpressed in a high percentage (up to 95%) of cervical cancers, releasing monomethyl auristatin E to disrupt microtubules and induce apoptosis (15, 16). In the pivotal innovaTV 204 trial, tisotumab vedotin produced an objective response rate of 24%, median progression-free survival of 4.2 months, and overall survival of 12.1 months, with tolerable toxicities such as ocular events and peripheral neuropathy (11, 14). Due to severe ocular risks, it includes a boxed warning, and peripheral neuropathy requires vigilant monitoring. Ongoing trials like innovaTV 205 and innovaTV 301 evaluate combinations with carboplatin, bevacizumab, or pembrolizumab, achieving objective response rates up to 55% in first-line contexts (11, 14).

Sacituzumab govitecan, targeting trophoblast cell-surface antigen 2, has demonstrated potential in pretreated recurrent or metastatic cervical cancer. A phase II trial in China reported an objective response rate of 50% overall, rising to 48% in patients previously exposed to immunotherapy (17). Trastuzumab deruxtecan, an anti-human epidermal growth factor receptor 2 conjugate, shows activity in expressing tumors; the DESTINY-PanTumor02 study noted an objective response rate of 37.1% across solid tumors, with approximately 50% in the cervical cancer subgroup of 40 patients (18, 19). Additional conjugates, such as mirvetuximab soravtansine (folate receptor alpha-targeted) and investigational agents like bulumtatug fuvedotin (Nectin-4-targeted), are under evaluation, signaling a move toward histology-agnostic paradigms (11, 14, 18).

Key challenges encompass resistance mechanisms, tumor heterogeneity, and adverse effects like neutropenia and ocular toxicity, demanding biomarker-guided approaches and synergies with immune checkpoint inhibitors (11, 14, 20). Preclinical evidence supports additive benefits; for instance, conjugate-induced depletion of vascular endothelial growth factor A-positive tumor-associated neutrophils can enhance programmed death-1 blockade (14, 20).

This bibliometric analysis delineates the progression of immunoconjugates in cervical cancer research from 2015 to 2024, pinpointing trends, focal areas, and unmet needs. Through examination of 244 publications, it seeks to clarify developmental paths, promote cross-disciplinary partnerships, and inform subsequent research to refine antibody-drug conjugates for better outcomes in this vulnerable patient group.

## 2 Materials and methods

### 2.1 Data source and search strategy

We conducted a systematic literature search on October 4, 2025, for publications from 2015 through 2024. The search targeted the Web of Science Core Collection (Science Citation Index Expanded, WoSCC) and the Scopus database. In WoSCC, we queried the Topic, Title, and Abstract fields; in Scopus, we queried Title, Abstract, and Keywords. The search strategy combined terms related to cervical cancer and immunoconjugates or antibody-drug conjugates. 

**Figure 1:**
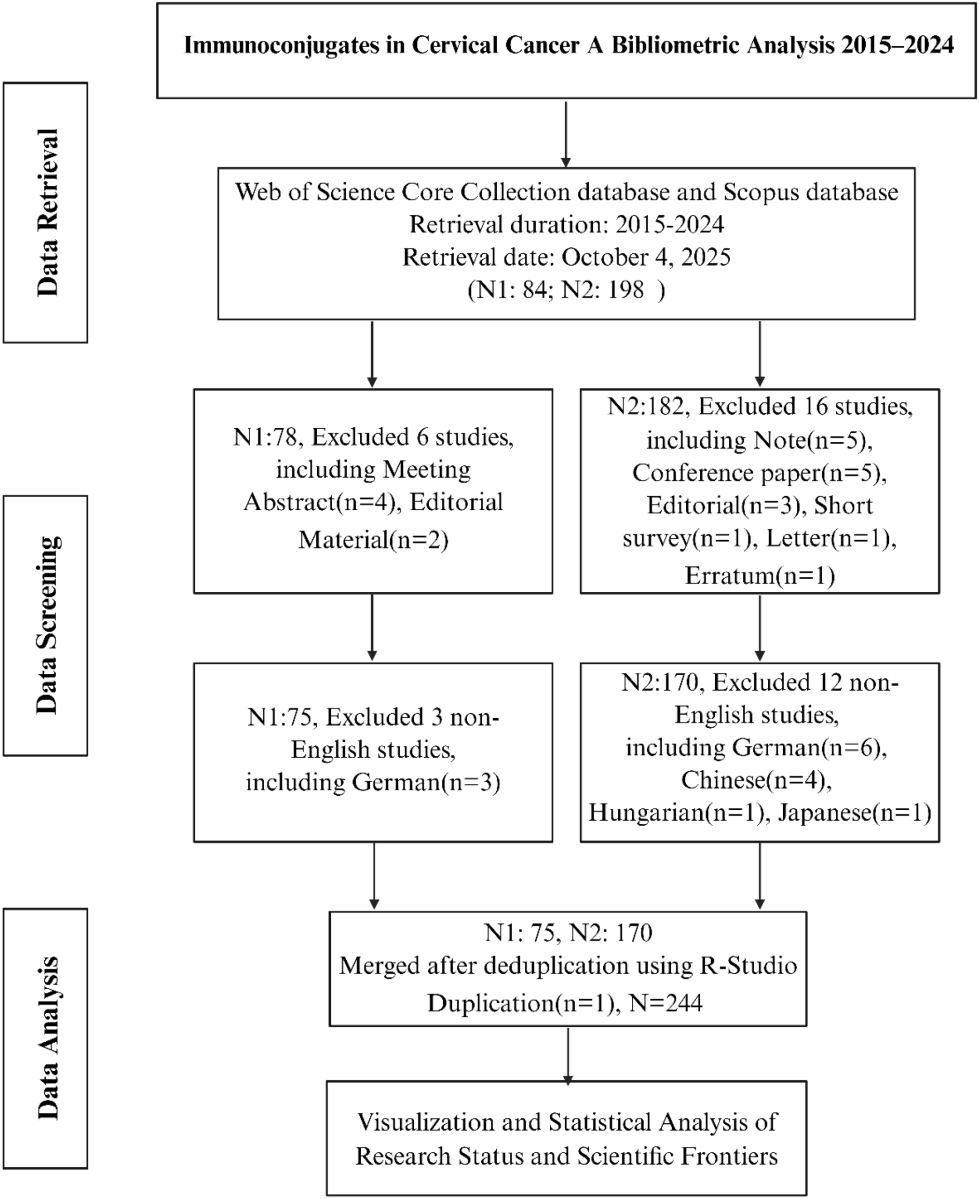
PRISMA-inspired study selection flowchart. PRISMA-style flow diagram showing identification, screening, eligibility assessment, and inclusion of publications on immunoconjugates in cervical cancer. Records were retrieved from Web of Science Core Collection (SCI-EXPANDED) and Scopus for the period 2015–2024 (search run October 4, 2025). Boxes report the number of records at each step, reasons for exclusion (e.g., non-research item types, non-English articles), and the final number of included publications (N = 244) used for bibliometric analyses.

Records were restricted to English-language original research articles and reviews. We excluded non-research items, such as editorials, letters, and meeting abstracts, as well as duplicates. Two reviewers independently screened the records and resolved discrepancies through discussion. Following de-duplication, we included 244 publications for quantitative and visualization analyses. **Figure 1** presents the PRISMA-style flowchart of study selection.

The search encompassed publications up to December 31, 2024. Although a few early-2025 publications appear in the references due to indexing delays, these primarily address data up to 2024 and were thus retained.

### 2.2 Bibliometric analysis and visualization

We merged data from WoSCC and Scopus, then processed them using R version 4.5.1 with the bibliometrix package (21, 22). Key bibliometric indicators were calculated, including annual publication counts, authorship metrics, and citation counts. For network construction and visualization, we employed VOSviewer version 1.6.20 to map keyword co-occurrence networks and identify thematic clusters (23–25). CiteSpace version 6.4.R1 was used to detect citation bursts in keywords and references (26–28). All graphs and networks were generated with publication-ready resolution and formatting.

## 3 Results

### 3.1 Publication output and growth trends

**Figure 2:**
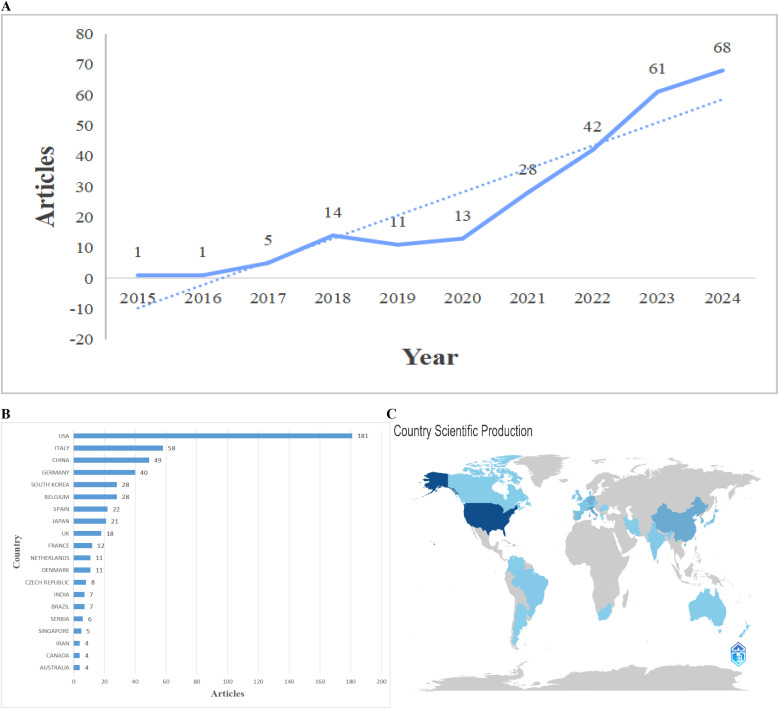
Annual publication trends and geographic distribution of research on immunoconjugates in cervical cancer (2015–2024). **(A)** Annual number of publications by year (solid line). The dashed line indicates the fitted trend/projection used to illustrate the overall growth pattern across the study period. **(B)** Country-level scientific production (publication counts) for the leading contributing countries; counts are adjusted for multi-country affiliations (fractional counting) where applicable. **(C)** World map of scientific production; color intensity reflects publication volume, with darker shading indicating higher output.

From 2015 to 2024, we identified 244 relevant publications across 118 journals. This period exhibited a substantial increase in research output, with a compound annual growth rate of 59.81 percent, indicating escalating interest in immunoconjugates for cervical cancer. Annual publication counts rose from one in 2017 to five in 2018 and 14 in 2019, followed by a modest decline to 11 in 2020. Output accelerated thereafter, with marked increases from 2021 to 2024 (**Figure 2A**). This surge after 2021 aligns with major clinical advancements, such as the FDA approval of tisotumab vedotin in 2021, signaling a shift from preclinical studies to clinical applications.

**Figure 3:**
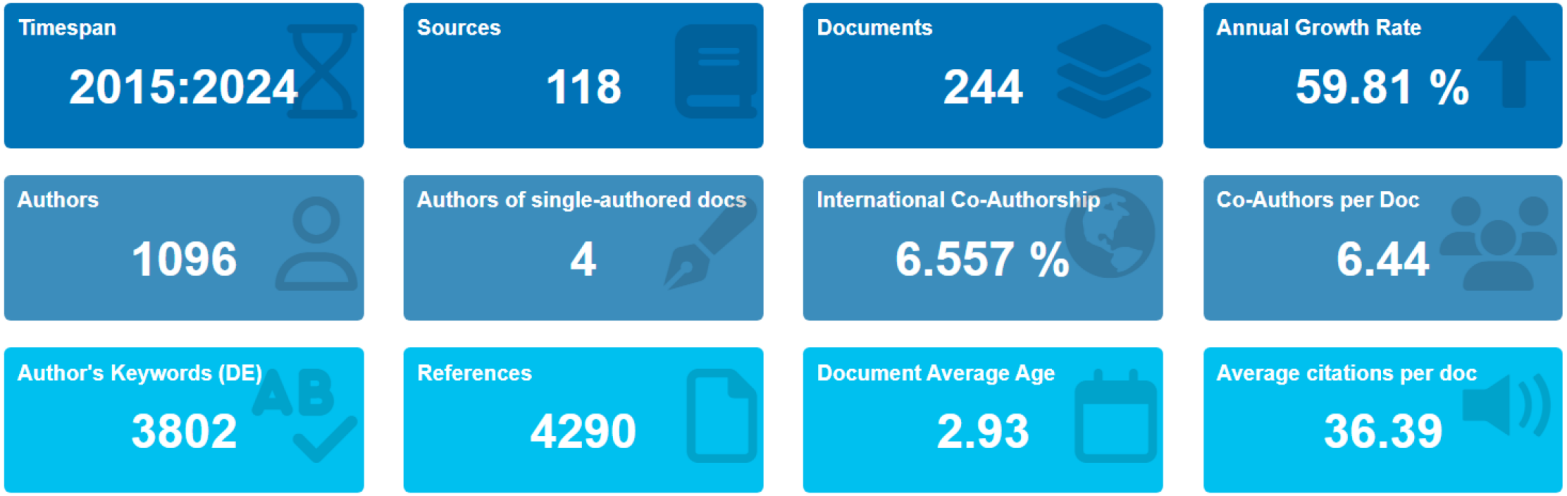
Overview of bibliometric research profile (2015–2024). Infographic summarizing the main descriptive metrics of the included dataset (N = 244), including the number of sources/journals, annual growth rate, number of unique authors, collaboration indicators (single-authored documents, average co-authors per document, and international co-authorship rate), and corpus-level characteristics (author keywords, total references, average document age, and average citations per document).

A total of 1,096 unique authors contributed, with an average of 6.44 co-authors per document, highlighting the collaborative character of the field. Only four documents, or 1.6 percent, were single-authored. International co-authorship occurred in 6.56 percent of documents, suggesting moderate global engagement. The limited extent of cross-country collaborations points to opportunities for enhanced international partnerships (**Figure 3**).

Geographically, the United States dominated with 181 publications, attributable to its robust biotechnology sector and investments in antibody-drug conjugate research (**Figure 2B**). Italy, China, and Germany ranked among the leading contributors, underscoring broadening global involvement. Contributions from low- and middle-income countries remained sparse, despite the disproportionate cervical cancer burden in these areas. **Figure 2C** depicts a world map of publication productivity, with countries shaded by publication volume from 2015 to 2024. A gradient from light to dark denotes increasing output.

### 3.2 Thematic clusters in keyword analysis

**Figure 4:**
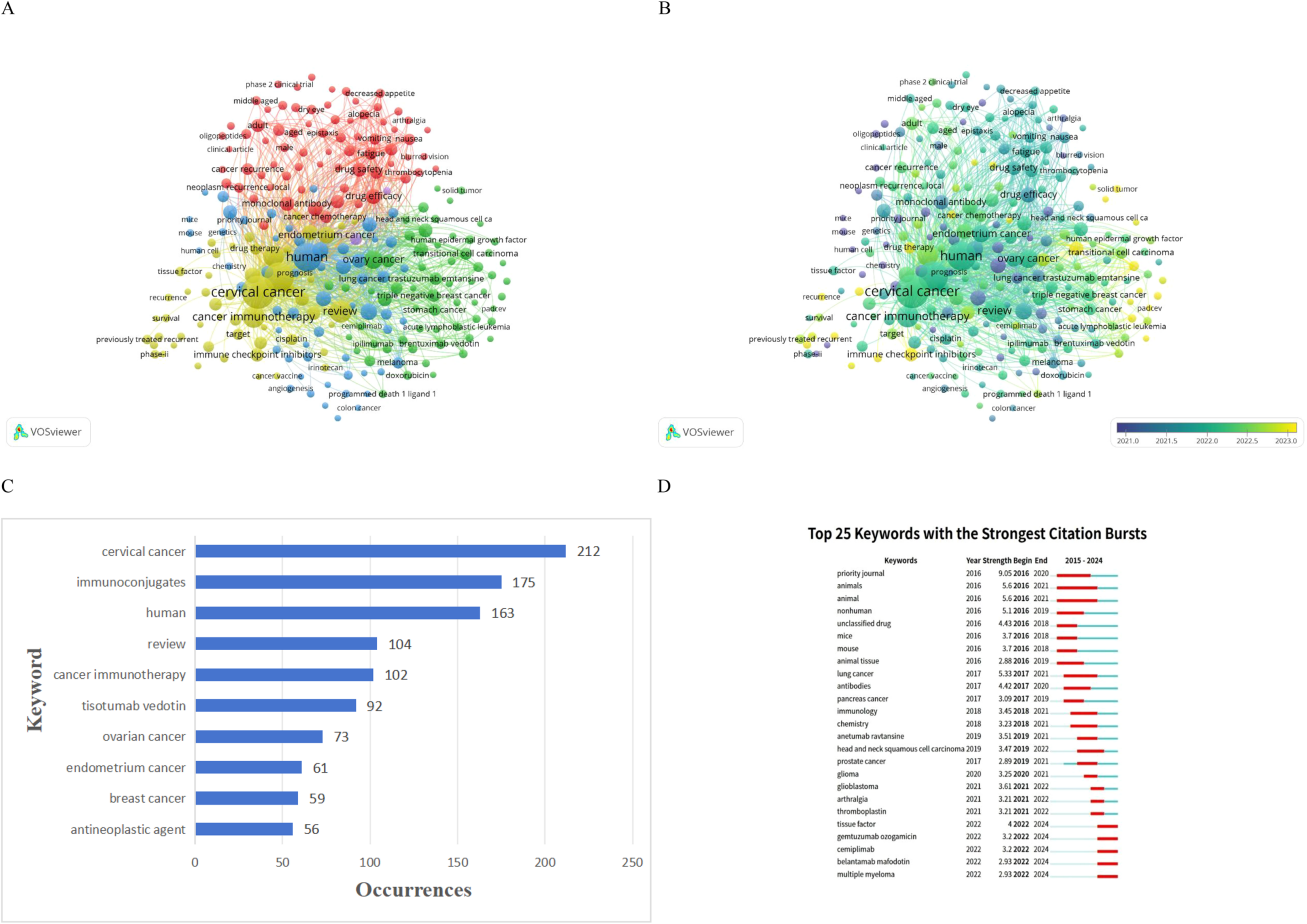
Keyword landscape and temporal dynamics in immunoconjugate research for cervical cancer (2015–2024). Analyses were performed using VOSviewer (co-occurrence networks) and CiteSpace (burst detection). **(A)** Keyword co-occurrence network of terms meeting the minimum occurrence threshold (≥10). Node size represents keyword frequency; links indicate co-occurrence strength. Colors denote clusters/themes: red (clinical safety/toxicity), green (hematologic malignancies/related ADC themes), blue (preclinical mechanisms), yellow (gynecologic oncology/targeted ADC applications), and purple (clinical trial design/outcomes). **(B)** Overlay visualization of the same network colored by average publication year (cooler colors = earlier; warmer colors = more recent), showing the temporal shift of hotspots. **(C)** Bar chart of the top 10 most frequent keywords in the dataset. **(D)** Top keywords with the strongest citation bursts identified by CiteSpace (γ = 0.8; minimum burst duration = 2 years); red bars indicate burst periods along the 2015–2024 timeline.

Keyword co-occurrence analysis delineated five thematic clusters (**Figure 4A**). These encompass clinical safety and toxicity management, including terms such as drug safety and adverse events; hematologic malignancies, with translational overlaps to blood cancer research; preclinical mechanisms, involving laboratory terms like animals and chemistry; gynecologic oncology, focusing on cervical cancer-specific topics such as combinations with immune checkpoint inhibitors and chemoradiotherapy; and trial design and emerging therapeutics, incorporating novel targets and methodologies. In **Figures 4A**, each cluster appears in a unique color, with a legend labeling them as Cluster 1 for clinical safety, Cluster 2 for hematologic malignancies, Cluster 3 for preclinical mechanisms, Cluster 4 for gynecologic oncology, and Cluster 5 for trial design. Keywords connect via lines denoting co-occurrence; thicker lines signify stronger or more frequent associations.

The network in **Figure 4A** demonstrates interconnected clusters. For example, tisotumab vedotin from the gynecologic oncology cluster links to drug safety in the clinical safety cluster, illustrating integrated attention to efficacy and adverse effects.

Early in the period, Clusters 2 and 3 predominated, featuring generic preclinical terms like animals and chemistry, which emphasized laboratory investigations. In later years, Clusters 4 and 1 gained prominence, with clinical and gynecologic oncology topics such as tissue factor and pembrolizumab becoming central.

The overlay visualization in **Figure 4B** colors keywords by average publication year, with blue for earlier periods and yellow for recent ones. This reveals a progression: research from 2015 to 2018, shown in blue, centered on preclinical themes in Clusters 2 and 3, exemplified by animals. In contrast, post-2021 studies, in yellow, prioritized clinical and gynecologic applications in Clusters 4 and 1, such as tissue factor and pembrolizumab. This evolution reflects a transition from basic laboratory work to clinical studies, propelled by antibody-drug conjugate approvals and trial outcomes.

**Figure 4C** presents a bar chart of the top 10 most frequent keywords, with keywords on the x-axis and occurrence counts on the y-axis. Cervical cancer appeared 212 times, and antibody-drug conjugate 181 times, affirming their core role. Other prevalent terms included cancer immunotherapy with 102 occurrences and targeted therapy, indicating integration with wider immunotherapy and precision medicine.

CiteSpace burst detection identified 25 keywords with the strongest citation bursts (**Figure 4D**). These bursts denote rapid increases in citation frequency, signaling emerging trends. **Figure 4D** is a timeline chart spanning 2015 to 2024 on the x-axis, with each keyword as a horizontal line; red segments mark burst periods. Early bursts from 2016 to 2018 focused on preclinical aspects, such as animals with a burst strength of 5.6 from 2016 to 2021, and mouse with 3.7 from 2016 to 2018, consistent with initial explorations in models. Mid-term bursts from 2017 to 2021 shifted to specific areas like lung cancer with 5.33 from 2017 to 2021, and immunotoxin with 3.45 from 2017 to 2021, highlighting applications to other solid tumors and immunotoxin therapies. A prominent mid-term burst involved anetumab ravtansine with 3.51 from 2019 to 2021, an antibody-drug conjugate targeting mesothelin, evidencing cross-influences from other cancers.

Recent bursts from 2022 to 2024 emphasize clinical translation. Tissue factor, with strength 4.0 from 2022 to 2024, coincided with successes in tissue factor-targeted therapy using tisotumab vedotin. Other late bursts included gemtuzumab ozogamicin with 3.2 from 2022 to 2024, and multiple myeloma with 2.93 from 2022 to 2024, implying influences from hematologic malignancy advances on cervical cancer research. Overall, these trends depict maturation from foundational science to targeted therapies and clinical terminology. By 2023 to 2024, emphasis intensified on clinical safety, efficacy, trial-related terms, adverse events, and combination strategies like antibody-drug conjugates with immune checkpoint inhibitors.

### 3.3 Citation analysis and influential studies

**Table 1** enumerates the top 10 most-cited articles, representing foundational contributions. These publications, each exceeding 180 citations, primarily report clinical trials, reviews on drug development, or translational antibody-drug conjugate research. The average citations per top-10 article reached 285, denoting considerable impact. Themes include pivotal trials, mechanisms of action, and antibody-drug conjugate development across tumor types, including histology-agnostic strategies.

**Table 1:** Top 10 Most cited articles in immunoconjugates for cervical cancer research (2015–2024).

Rank	Article title	Publication year	Journal	Impact factor (2024)	First author	Total citations	TC per year
1	Efficacy and Safety of Trastuzumab Deruxtecan in Patients With HER2-Expressing Solid Tumors: Primary Results From the DESTINY-PanTumor02 Phase II Trial (18).	2024	Journal of Clinical Oncology	41.9	Funda Meric-Bernstam	502	251
2	Efficacy and safety of tisotumab vedotin in previously treated recurrent or metastatic cervical cancer (innovaTV 204/GOG-3023/ENGOT-cx6): a multicentre, open-label, single-arm, phase 2 study (11).	2021	The Lancet Oncology	35.9	Robert L. Coleman	327	65.4
3	Targeting B7-H3 (CD276) with an Eradication of Tumors through Simultaneous Ablation of CD276/B7-H3-Positive Tumor Cells and Tumor Vasculature (29).	2017	Cancer Cell	44.5	Deryk Loo	326	36.22
4	Antibody-drug conjugates: Smart chemotherapy delivery across tumor histologies (30).	2021	CA: A Cancer Journal for Clinicians	232.4	Jacob J. Adashek	311	77.75
5	Targeting HER2 with Trastuzumab Deruxtecan: A Dose-Expansion, Phase I Study in Multiple Advanced Solid Tumors (31).	2020	Cancer Discovery	33.3	Junji Tsurutani	301	50.17
6	Sacituzumab govitecan, a Trop-2-directed antibody-drug conjugate, for patients with epithelial cancer: final safety and efficacy results from the phase I/II IMMU-132–01 basket trial (32).	2021	Annals of Oncology	65.4	Aditya Bardia	284	56.8
7	The emergence of trophoblast cell-surface antigen 2 (TROP-2) as a novel cancer target (33).	2018	Oncotarget	1.6	David M. Goldenberg	248	31
8	Mechanisms of ADC Toxicity and Strategies to Increase ADC Tolerability (34).	2023	Cancers	4.4	Toan D. Nguyen	212	70.67
9	Monoclonal antibody therapy of solid tumors: clinical limitations and novel strategies to enhance treatment efficacy (35).	2019	Biologics: Targets and Therapy	3.4	Esteban Cruz	203	29
10	Tisotumab vedotin in patients with advanced or metastatic solid tumours (innovaTV 201): a first-in-human, multicentre, phase 1–2 trial (36).	2019	The Lancet Oncology	35.9	Johann S de Bono	187	26.71

**Table 1** ranks the top 10 most cited publications within the merged bibliometric dataset on immunoconjugates in cervical cancer (2015–2024). Total Citations (TC) and TC per Year were calculated from the deduplicated records retrieved from Web of Science Core Collection (SCI-EXPANDED) and Scopus (search run October 4, 2025); TC per Year reflects time-normalized citation impact. Impact Factor (2024) values were taken from the 2024 Journal Citation Reports (JCR) for the corresponding journals. Citation-based indicators reflect publication and citation behavior within the indexed literature and should not be interpreted as direct measures of clinical efficacy or study quality. TC, total citations; JCR, Journal Citation Reports.

The most cited article, by Meric-Bernstam and colleagues in 2024, presented primary results from the DESTINY-PanTumor02 Phase II trial (18). This study showed efficacy of trastuzumab deruxtecan in HER2-expressing solid tumors, with an overall response rate of 37.1 percent, including cervical cancer subgroups (18). It established proof-of-concept for HER2-targeted treatment in cervical cancer, extending beyond breast and gastric cancers (18). The high citations per year, approximately 251, highlight its recent and transformative influence on histology-agnostic approaches (18).

The second most cited, by Coleman and colleagues in 2021, described the innovaTV 204 trial, which supported FDA approval of tisotumab vedotin (11). It reported a 24 percent overall response rate in heavily pretreated patients and detailed toxicities, including ocular effects requiring prophylactic care and peripheral neuropathy (11). With 327 citations and about 82 per year, this work illustrates the role of regulatory-approving trials in guiding the field (11).

Other influential articles offer aligned insights:

Loo and colleagues in 2017 conducted a preclinical study on B7-H3-targeted antibody-drug conjugates (29). It showed that targeting B7-H3-positive tumor cells and vasculature yields potent antitumor effects (29). This foreshadowed ongoing development of B7-H3 conjugates, with early clinical data from DB-1311/BNT324 indicating around 43 percent overall response rate in advanced cervical cancer, affirming translational relevance (29).

Adashek and colleagues in 2020 reviewed histology-agnostic antibody-drug conjugate development, advocating molecular targeting independent of histology (30). This supports exploration of HER2-targeted therapies like trastuzumab deruxtecan in cervical cancer (30).

Tsurutani and colleagues in 2020 reported a Phase I trial of trastuzumab deruxtecan in advanced solid tumors, with a 28.3 percent confirmed overall response rate (31). Although one cervical cancer patient showed no response, the study emphasized interstitial lung disease risks, informing subsequent monitoring (31).

Bardia and colleagues in 2021 evaluated sacituzumab govitecan, a Trop-2-directed conjugate, in epithelial cancers, achieving a 22.2 percent overall response rate in gynecologic cohorts (32). Combined with Goldenberg and colleagues in 2018, who identified Trop-2 as a target in solid tumors, these underpin sacituzumab govitecan investigations in cervical cancer (33). A Phase II trial in China reported 43 percent overall response rate, rising to 48 percent in PD-1 inhibitor-pretreated patients, bolstering Trop-2 targeting (17).

Nguyen and colleagues in 2023 analyzed antibody-drug conjugate toxicities and mitigation, relevant to the clinical safety cluster (34). It addresses neutropenia in about 42 percent of patients, advocating monitoring and dose adjustments (34).

Cruz and Kayser in 2019 reviewed monoclonal antibody therapies in solid tumors, discussing efficacy enhancements like improved tumor penetration (35). This applies to antibody-drug conjugates, emphasizing combination strategies to overcome microenvironment barriers, as seen in our analysis (35).

These studies collectively delineate milestones, from epidemiological rationale to clinical validation in cervical cancer.

**Figure 5:**
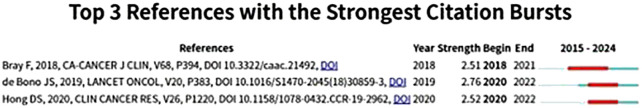
Reference citation burst timeline for the top 3 burst references (2015–2024). Timeline visualization of the three references with the strongest citation bursts detected by CiteSpace (γ = 0.8; minimum burst duration = 2 years). Each row corresponds to one reference; the red segment denotes the burst interval (years in which citations increased sharply) over the 2015–2024 baseline, highlighting shifts from epidemiological foundations to key clinical milestones relevant to antibody–drug conjugates in cervical cancer.

**Figure 5** timelines the three strongest reference citation bursts. The top burst, for de Bono and colleagues in 2019, followed the first-in-human tisotumab vedotin trial, showing safety and preliminary efficacy (36). The second, for Bray and colleagues in 2018, provided global cancer statistics, noting 18.1 million new cases and cervical cancer’s fourth rank in women (1). The third, for Hong and colleagues in 2020, reported a multi-cohort tisotumab vedotin trial with 24 percent overall response rate in recurrent disease (16). These bursts demonstrate how epidemiology motivated research, and early trials validated antibody-drug conjugates, propelling further efforts.

## 4 Discussion

Our bibliometric analysis of 244 publications from 2015 to 2024 reveals growing interest in immunoconjugates for cervical cancer. The exponential increase in publications, especially after 2021, aligns with key clinical developments, including FDA approval of tisotumab vedotin and promising results from other antibody-drug conjugate trials. This pattern indicates a swift shift from preclinical investigations to clinical validation in recent years.

Contributions predominantly originate from high-resource countries, with the United States leading, followed by Italy, China, and Germany. This distribution reflects established research infrastructure and investments in novel therapeutics. Conversely, limited output from low-resource regions highlights a discrepancy between areas of highest cervical cancer burden and concentrations of research activity. Only 6.56 percent of documents in our dataset featured international co-authorship, which suggests constrained global collaboration in this field. This modest level of cross-border engagement presents opportunities to expand international research networks. For example, multinational clinical trials or research consortia could facilitate knowledge exchange and extend antibody-drug conjugate advancements to varied populations.

Keyword analysis illustrates thematic maturation over time. Early research from 2015 to 2018 emphasized preclinical aspects, such as mechanisms and targets of antibody-drug conjugates. In later years, clinical studies on safety and efficacy gained prominence. This transition appears in the emergence of keywords like tissue factor and pembrolizumab from 2022 to 2024, which signify integration of antibody-drug conjugates with immunotherapy. The overlay visualization in **Figure 4B** demonstrates how preclinical terms receded as clinical ones advanced. By 2022, discussions of trial outcomes, combination therapies, and adverse effect management became central, corresponding to the field’s progression toward practical application.

Results also underscore the interdisciplinary character of antibody-drug conjugate research in cervical cancer. Interconnected clusters, spanning preclinical, clinical, and safety domains, indicate that advancements often stem from integrated perspectives. For instance, insights from hematologic malignancy studies on payload toxicity or resistance inform cervical cancer trials. In turn, clinical observations in cervical cancer, such as managing tissue factor-targeted therapy toxicities, offer broader applicability. Connections in **Figures 4A**, including those between gynecologic oncology and drug safety, show researchers balancing efficacy and tolerability, essential for clinical translation. .

Influential papers identified in our analysis support these findings. Top-cited works by Meric-Bernstam and colleagues, and by Coleman and colleagues, delivered clinical evidence for antibody-drug conjugates in cervical cancer (11, 18). These trials confirmed meaningful responses through targeting HER2 or tissue factor, establishing new treatment frameworks. Additional studies, such as those by Loo and colleagues, and by Goldenberg and colleagues, broadened potential targets with B7-H3 and Trop-2, now under clinical evaluation (29, 33). Analyses by Nguyen and colleagues addressed safety strategies, aligning with the field’s focus on improving tolerability, for example by preventing interstitial lung disease with trastuzumab deruxtecan or managing neutropenia with sacituzumab govitecan (34). Consistency between these high-impact publications and our bibliometric trends affirms that the analysis captures the field’s primary direction.

Several limitations warrant consideration. Our study drew from two major databases, Web of Science Core Collection and Scopus, and restricted to English-language publications. This approach may overlook non-English works or those not indexed, including certain conference proceedings or regional reports. Future analyses could incorporate additional sources, such as PubMed or Google Scholar, and multilingual literature for a more comprehensive view, in line with suggestions from reviewers. Bibliometric metrics, including citation counts and bursts, indicate research focus and popularity but do not assess clinical outcomes or patient benefits directly. A highly cited preclinical study, for instance, may not yield effective therapies. We contextualized these metrics yet recognize their role as proxies for influence. We did not conduct formal co-authorship or country collaboration network analyses, as requested by a reviewer. Although we quantified international co-authorship and discussed collaboration qualitatively, social network methods could better depict institutional and national interactions. Subsequent studies might apply such techniques to visualize partnerships. Finally, reliance on published literature may exclude late 2024 to 2025 trial data or conference presentations, though we incorporated relevant updates where possible, such as emerging B7-H3 antibody-drug conjugate findings.

Future directions emphasize overcoming persistent challenges in antibody-drug conjugate therapy for cervical cancer. Combination regimens represent a key focus, with antibody-drug conjugates increasingly paired with immune checkpoint inhibitors or anti-angiogenic agents. Keyword bursts and cluster patterns, such as pembrolizumab alongside antibody-drug conjugate terms, indicate rising interest in these synergies. Trials like innovaTV 205, where tisotumab vedotin with pembrolizumab achieved a 41 percent overall response rate in first-line recurrent or metastatic cervical cancer, suggest potential efficacy gains. Biomarker optimization is another priority, to identify responsive patients. Refining HER2 expression thresholds for trastuzumab deruxtecan, or predictive markers for sacituzumab govitecan response or toxicity, will support personalized approaches. The target pipeline continues to grow, with Nectin-4, B7-H3, and others in early trials, potentially benefiting patients lacking expression of established targets like tissue factor or HER2.

Global collaboration and equity are essential for progress. Cervical cancer disproportionately affects resource-limited settings, yet most antibody-drug conjugate research occurs in high-income countries. Addressing this requires deliberate actions, including diverse trial populations, technology transfer partnerships, and affordable treatments. Our observation of approximately 6.6 percent international co-authorship highlights the need for inclusive networks. Collaborations between high-burden regions and advanced centers can align innovations with global needs. Integrating prevention, through HPV vaccination and screening, with therapeutic developments remains critical, as emphasized in global cancer statistics reports noting ongoing disparities.

In summary, this bibliometric analysis establishes antibody-drug conjugates as central to cervical cancer therapy evolution. Surging research output and citations reflect enthusiasm for agents like tisotumab vedotin and trastuzumab deruxtecan, alongside efforts to resolve obstacles via innovation. Sustained advancement, through new targets, combinations, refined selection, and inclusive collaboration, will shape realization of immunoconjugate potential. Our depiction of the field’s trajectory offers guidance for researchers, clinicians, and policymakers to prioritize impactful domains, with the goal of enhancing outcomes for this underserved population.

## Data Availability

Publicly available datasets were analyzed in this study. This data can be found here: The datasets analyzed in this study were retrieved from the Web of Science Core Collection (SCI-EXPANDED) and Scopus databases, as detailed in the Methods section (Section 2.1). The raw bibliographic data, including search queries, publication records, and citation information, are publicly accessible through these subscription-based databases using the provided search strategy. Deduplicated and merged datasets were processed using open-source R software (v4.5.1) with the bibliometrix package. Visualization files generated via VOSviewer (v1.6.20) and CiteSpace (v6.4.R1) are available upon reasonable request from the corresponding author. No new primary data were generated or analyzed in this bibliometric study.
